# Inhibitory effect of lingonberry extract on HepG2 cell proliferation, apoptosis, migration, and invasion

**DOI:** 10.1371/journal.pone.0270677

**Published:** 2022-07-08

**Authors:** Liangyu Zhu, Yandong Zhang, Yongchun Li, Hua Wang, Guang Shen, Zhenyu Wang

**Affiliations:** 1 School of Forestry, Northeast Forestry University, Harbin, China; 2 Heilongjiang Academy of Sciences Institute of Natural Resources and Ecology, Harbin, China; 3 School of Food Science and Engineering, Harbin Institute of Technology University, Harbin, China; 4 College of Chemistry and Life Science, Chifeng University, Chifeng, China; Huazhong University of Science and Technology, CHINA

## Abstract

Lingonberry (*Vaccinium vitis-idaea* L.) extract contains various active ingredients with strong inhibitory effects on cancer cell growth. HepG2 cells were treated with various concentrations of lingonberry extract, cell inhibition rate was measured by CCK-8 assay, and apoptosis rate by annexin-propidium iodide double-staining assay. The cell cycle was analyzed by flow cytometry, and cell migration and invasion by transwell assay. Real-time reverse transcription-PCR and western blotting were employed to analyze the expression of C-X-C motif chemokine ligand 3 (CXCL3). Ki-67, TUNEL, and transwell assays were used to verify the relationship between CXCL3 expression and cell proliferation, apoptosis, migration, and invasion. The composition of lingonberry extract was: 37.58% cyanidin-3-O-glucoside, 10.96% kaempferol 3-O-arabinoside, 4.52% epicatechin, 4.35% chlorogenic acid, 3.83% catechinic acid, 1.54% isoquercitrin, 1.05% 4-hydroxycinnamon acid, 1.03% cyanidin chloride, 0.85% 2,3-dihydroxybenzoic acid, 0.55% quercetin, 0.36% D-(-)-quininic acid, 0.96% caffeic acid, 0.16% ferulic acid, 0.12% oleanolic acid, and 0.03% ursolic acid. Lingonberry extract inhibited the proliferation of HepG2 cells in a dose-dependent manner. After 48 h exposure to 100 μg/mL extract the inhibition rate and IC_50_ were 80.89±6.05% and 22.62 μg/mL, respectively. Lingonberry extract promoted late apoptosis in HepG2 cells and arrested the cell cycle at G2/M and S phases. Lingonberry extract also promoted the apoptosis of HepG2 cancer cells, inhibiting their proliferation, migration, and invasion by regulating the expression of CXCL3. This study offers new insight into the antihepatoma activity of lingonberry extract and provides a basis for the development of pilot antitumor drugs.

## Introduction

Cancer is a major cause of death in humans and hepatocellular carcinoma is one of the most common fatal malignancies. A large number of people die of hepatomas each year and the prevalence of hepatomas is increasing year-on-year. China has the highest clinical incidence of hepatomas among Asian countries [1, 2]. However, the efficiency of hepatoma treatments is far from satisfactory. The development of novel and effective drug treatments and research into new treatment methods are key to lowering increasing hepatoma-related mortality.

It has been demonstrated that natural compounds extracted from plants, vegetables and fruits can prevent cancer development and reduce tumor growth [3, 4]. Natural extracts from sugarcane have been shown to inhibit growth of human lung cancer A549 cells and human ovarian cancer SKOV-3 cells [5]. Cranberry juice has an antiproliferative effect on cervical cancer cells (HeLa), exhibiting a significant difference compared to a control group at concentrations above 0.125 mg/mL [6]. At 1.0 mg/mL, bog bilberry (*Vaccinium uliginosum* L.) strongly inhibits the viability of colon cancer cells (Caco-2) and hepatoma cells (HepG2) treated for 72 h [7]. Cactus extracts suppress the proliferation of human breast cancer cell lines MCF-7 and MDA-MB-231 by inducing G2/M cell cycle arrest and apoptosis [8]. The extracts of Tribulus terrestris fruit and Xanthium strumarium fruit had differential effects on cell proliferation of oral cancer cells. In addition, the fruit extracts hampered cell migration and invasion of oral cancer cells [9].

C-X-C motif chemokine ligand 3 (CXCL3) is a single-stranded protein of the CXC chemokine family. It activates C-X-C motif chemokine receptor 2 (CXCR2), initiating downstream signal activation and regulating cell migration and invasion. The CXCL3/CXCR2 signaling pathway is associated with a variety of diseases [10, 11] and is important in tumor development and progression. Overexpression of CXCL3 can increase the risk of prostate cancer tumor formation and plays multiple roles in the progression and metastasis of this cancer [12]. In non-small-cell lung cancer (NSCLC), circMET promotes proliferation, metastasis, and immune evasion by regulating Mir-145-5p/CXCL3 [13]. CXCL3 overexpression promotes the oncogenic potential of uterine cervical cancer cells through the MAPK/ERK pathway [14]. Cancer cell-derived sST2 upregulates CXCL3 by inhibiting IL-33-ST2L signaling in the pancreatic cancer microenvironment and enhances tumor growth [15]. There has been little research into whether CXCL3 is expressed in hepatocellular carcinoma, and more work is needed to verify if CXCL3 overexpression is a potential tumor marker.

Lingonberry (*Vaccinium vitis-idaea* L.) is a plant of the azalea family. *Vaccinium* are found in northeast China, Russia’s alpine region, the Korean Peninsula, North America, and northern Europe [3, 16]. Lingonberry fruit are considered a natural, safe food eaten whole or processed, since they contain a variety of health-promoting functional ingredients. The biological and antioxidant activities of phenolic compounds in lingonberry fruit have been studied [17–19]. Lingonberry exhibits antidiabetic properties in obese mice [20], while lingonberry anthocyanin protects myocardial cells from oxidative stress-induced apoptosis [21]. It inhibits the expression of inflammatory cytokines [22], inhibits tumorigenesis and cancer cell growth, and has antiproliferative effects on the digestive tract [23]. Fermented lingonberry juice significantly weakens the proliferation and invasion of SCC-25 human oral cancer cells [24].

The antihepatoma mechanisms of functional components of lingonberry have only recently been studied [25]. The present study examines the functional potential of lingonberry extracts by exposing human hepatoma HepG2 cells to various concentrations *in vitro*. The effects of the extracts on the proliferation, apoptosis, migration, and invasion of HepG2 cells and on CXCL3 expression levels are monitored, providing a theoretical basis for the inhibitory effects of lingonberry extracts on HepG2 proliferation.

## Materials and methods

### Reagents and instruments

Hepatoma cells (HepG2), colon cancer cells (DLD-1), and breast cancer cells (MCF-7) were provided by the Second Hospital of Harbin (China). All cell lines were mycoplasma-free and were authenticated using short tandem repeat (STR) genotyping within the last three years and were verified as being identical to STR profiles in comparable databases (Zhejiang Ruyao Biotechnology Science and Technology Company Limited). Dulbecco’s modified eagle medium (DMEM) with high glucose was sourced from Gibco (USA), Roswell Park Memorial Institute (RPMI) 1640 medium from BioReagent, and fetal bovine serum (FBS) from Invitrogen (USA). Penicillin-streptomycin and phosphate buffered saline (PBS) were purchased from Sigma and Gibco, respectively. Trypsin (0.25%) was obtained from HyClone (USA), and 96-well tissue culture plates from Corning (USA). Cell counting kit-8 (CCK-8) and cell cycle assay kits were purchased from Dojindo Laboratories (Japan) and Shanghai Ruian Biological Engineering Co. Ltd., respectively. AnnexinV-fluorescein isothiocyanate (FITC) and propidium iodide (PI) apoptosis assay kits were obtained from Beyotime Biotechnology. The Q Exactive and Ultimate 3000 UPLC systems were sourced from Thermo Fisher Scientific.

### Preparation of berry extracts

Lingonberry was crushed and extracted twice with 80% ethanol at a solid-liquid ratio of 1:15 (w/v). The extracts were combined and the solvent was removed by rotational evaporation. Extract powder was obtained by YWD07 macroporous resin purification, decompression concentration, and vacuum freeze drying. This was then dissolved in PBS and passed through a 0.22 μm filter membrane. Bog bilberry and frozen cranberry fruit extracts were also prepared using the above protocol.

### Cell culture

HepG2 and MCF-7 cells were cultured in DMEM containing 10% FBS. DLD-1 cells were cultured in RPMI 1640 with 10% FBS. All cell lines were grown in a 5% CO_2_ thermostatic incubator at 37°C.

### Cell Inhibition rate by CCK-8 assay

HepG2, MCF-7, and DLD-1 cells in the logarithmic growth phase were seeded into 96-well plates (1×10^4^ cells/well) and cultured overnight in a 5% CO_2_ thermostatic incubator at 37°C. Upon cell attachment, the medium was discarded and 200 μL of medium containing various concentrations of lingonberry, bog bilberry, or cranberry extract was added (20, 40, 60, 80 or 100 μg/mL). The same volume of medium was added to a control group. Six replicate wells were prepared at each concentration. After culturing for 48 h the medium was discarded. Fresh medium (100 μL) and CCK-8 assay solution (10 μL) were added and incubated for 2 h at 37°C. The absorbance at 490 nm was measured in each well using a microplate reader.

### Cell apoptosis rate by annexin-PI double staining assay

HepG2 cells in the logarithmic growth phase were digested using 0.25% trypsin, transferred to single-cell suspension in medium containing 10% FBS and inoculated into 24-well plates. Cell concentration was adjusted to 2×10^5^ cells/well, cultured for 24 h, and the original medium removed following cell attachment. Medium containing *Vaccinium vitis-idaea* L. extract (20, 40, 60, 80 or 100 μg/mL) was added to the HepG2 cells and cultured in 5% CO_2_ at 37°C for 48 h (an equal volume of DMEM was added to the control group). Suspended cells were harvested by centrifugation at 2000 r/min for 5 min. Adhered cells (1 to 5×10^5^ cells) were harvested by 0.25% trypsin digestion, washing with pre-cooled PBS, and re-suspension in 500 μL buffer. Cells were mixed with 5 μL Annexin V-FITC, stained for 20 min in the dark at room temperature, then mixed with 5 μL PI for 5–15 min in the same environment. Flow cytometry was performed within 1 h [8, 11].

### Cell cycle by flow cytometry

HepG2 cells were seeded into 6-well plates at a density of 1×10^5^ cells/well and cultured overnight. Lingonberry extract was added at 20, 40, 60, 80 or 100 μg/mL alongside the control group. After 48 h the cells were harvested using 0.25% trypsin, centrifugation at 1000 r/min for 5 min, washing twice in pre-cooled PBS, and fixing in 70% ice-cold ethanol at 4°C overnight. Cells were centrifuged again to remove ethanol, washed twice in PBS, incubated with fluorescent dye at room temperature in the dark for 30 min, and the cell cycle was detected by flow cytometry [25, 34].

### Cell migration and invasion by transwell assay

HepG2 cells were treated with lingonberry extract at 20, 40, 60, 80 or 100 μg/mL (no extract in the control group) for 48 h. Cells were seeded into the upper chamber at 2×10^4^ cells/mL while the lower chamber contained 600 μL of medium with 10% FBS. After 24 h the cells in the upper chamber were wiped off using cotton swabs, fixed in methanol for 30 min, and stained with 0.1% crystal violet for 10 min. After washing in PBS, the cells were observed under a microscope and immediately photographed in randomly-selected visual fields. For the cell invasion experiment, Matrigel glue was added to the upper layer of the chamber and polymerized at 37°C for 30 min. Excess liquid was absorbed and the upper chamber was air-dried on an ultra-clean stage for 1 h. HepG2 cells treated with extracts (20, 40, 60, 80, or 100 μg/mL) for 48 h were inoculated into the upper chamber at a density of 2×10^4^ cells/mL, and 600 μL medium containing 10% FBS was added to the lower chamber. Other procedures were as for the cell migration test [11, 13].

### CXCL3 expression in cancer cells by western blotting

HepG2 cells were treated with lingonberry extract (20, 40, 60, 80, or 100 μg/mL; zero in the control group) for 48 h. Radio-immunoprecipitation assay lysis buffer was added and the concentration of proteins was determined by bicinchoninic acid kit. SDS-PAGE was performed and the resulting protein samples were transferred onto a polyvinylidene fluoride membrane which was blocked with 5% skimmed milk powder at room temperature for 1 h. Samples were incubated with primary antibody at 4°C overnight and the membrane washed with tris-buffered saline-Tween 20 (TBST). Samples were then incubated with secondary antibody at room temperature for 2 h and washed three times with TBST. After exposure, images were developed in a dark room, immersed in fixation solution, and film was processed using Quantity One gel analysis software.

### CXCL3 expression in cancer cells by Real-time Reverse Transcription-Polymerase Chain Reaction (RT-PCR)

Total ribonucleic acids were extracted and identified. Complementary deoxyribonucleic acids (cDNAs) were synthesized as follows: mixtures were prepared in 0.2 mL RNase-free Eppendorf tubes and reacted at 37°C for 15 min, 85°C for 5 s, and 4°C for 60 min. The resulting cDNAs were diluted with 180 μL of ddH_2_O and stored at -20°C for second strand cDNA synthesis or PCR amplification. The primer sequences for quantitative PCR were as follows: β-actin-F GGCTGTGCTATCCCTGTACG, β-actin-R AGGTAGTCAGTCAGGTCCCG, CXCL3-F TGTGAATGTAAGGTCCCCCG, and CXCL3-R TTGGTGCTCCCCTTGTTCAG. The PCR program was set up for amplification and relative expression levels were calculated using 2^–ΔΔCt^.

### Cell transfection and grouping

A Lenti-Pac HIV Expression Packaging Kit (GeneCopoeia, China) was used for overexpression of CXCL3 and mock transfection of HepG2 cells in five groups [26]: control (no treatment), extract group (treated with 80 μg/mL lingonberry extract), negative control (NC; transfected with NC empty plasmid), overexpression group (OE-CXCL3; transfected with overexpressed CXCL3 plasmid), and extract with overexpression group (OE-CXCL3; treated with 80 μg/mL lingonberry extract and transfected with overexpressed CXCL3 plasmid). The expression of CXCL3 after transfection was determined by RT-PCR and Western blotting. The migration and invasion capabilities of the HepG2 cells were measured as described above [13, 14].

### Cell proliferation Ki-67 assay

The five HepG2 groups were fixed with 4% paraformaldehyde for 15 min, permeabilized with 0.2% Triton X-100 for 5 min, and blocked with 1% FCS for 1 h at room temperature. The cells were labeled with mouse anti-human Ki-67 monoclonal antibody at 1:50 dilution in 1% FCS, incubated at 4°C overnight, and stained with goat anti-mouse IgG (H+L) secondary antibody conjugated to Alexa Fluor Plus 488 at a dilution of 1:1000 for 45 min at room temperature. Nuclei were stained with 5 μg/mL final concentration of DAPI for 30 min. Samples were washed three times with PBS and cells were examined by fluorescence microscopy [27].

### Terminal deoxynucleotidyl transferase‑mediated dUTP nick end labeling (TUNEL) assay

The five HepG2 groups were placed in a 96-well plate at 1 x 10^5^ cells/well and cultured for 2 h before 50 μl TUNEL reaction solution was added and incubated for 60 min. After rinsing, conversion solution was added to the cells which were incubated 60 min, then stained with DAB for 30 min, and examined by light microscopy. Red granules in the nuclei indicated apoptotic cells [28].

### Lingonberry extract composition

An Ultimate 3000 UPLC system and Thermo Scientific Q Exactive were used for analysis of the composition of lingonberry extract. Chromatography parameters were as follows: Hypersil Gold 100×2.1 mm, 3 μm column (Thermo Scientific, Germany) at 25°C; 0.1% acetic acid in acetylene (mobile phase A) and 0.1% acetic acid in water (mobile phase B) with the gradient elution A = 5-20-90-90-5-5% at time 0-4-15-18-18.1–25 min; detection wavelength 280 nm; and flow rate 0.25 mL/min. An m/z mass spectrometry scan was performed under electrospray ionization.

### Statistical analysis

Statistical analyses were carried out using SPSS 17.0 software. Unpaired *t*-tests were applied to evaluate significant differences. Statistically significant differences were defined as *p* < 0.05 (*) and strongly significant differences as *p* < 0.01 (**). In cell culture experiments, culture bottles were randomly numbered and randomly selected. The cells in culture bottles were digested and counted on plates, corresponding to the experimental groups with various concentrations from left to right. All data are expressed as mean ± standard deviation of at least three independent experiments.

## Results

### Inhibitory effect of lingonberry on HepG2 proliferation

#### Inhibitory effect of lingonberry on proliferation of various cancer cells

**Fig 1:**
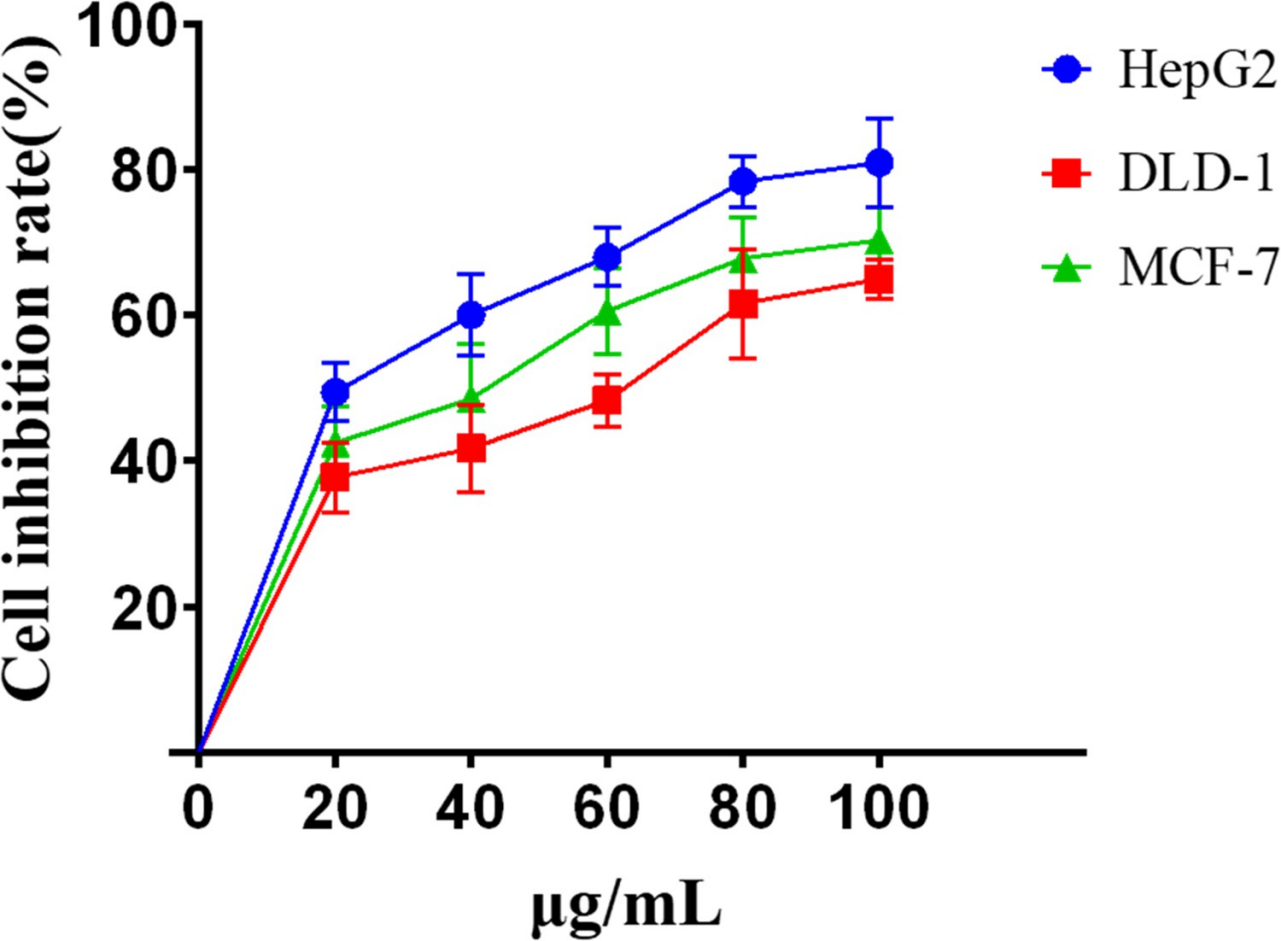
Inhibition rates of lingonberry extract on proliferation of various cancer cells.

CCK-8 assay results (Fig 1) show that increasing the concentration of lingonberry extract (48 h exposure) gradually increased its inhibitory effects on the cancer cells HepG2, DLD-1 and MCF-7.

At 80 μg/mL, lingonberry inhibited HepG2 cells by 78.34±3.55% with a half-maximal inhibitory concentration (IC_50_) of 22.62 μg/mL. DLD-1 cells were inhibited by 61.58±7.46% with an IC_50_ of 49.86 μg/mL. When the concentration was increased to 100 μg/mL, the inhibition rates of HepG2 and DLD-1 cells increased but were not significantly higher than at 80 μg/mL. At 100 μg/mL, inhibition rate was 80.89±6.05% for HepG2 cells and 64.93±2.64% for DLD-1 cells.

At 60 μg/mL, lingonberry exhibited an inhibition rate of 60.54±5.83% and an IC_50_ of 33.91 μg/mL with MCF-7 cells. At higher concentrations the inhibition rate increased, but not significantly above that at 60 μg/mL. At 100 μg/mL, the MCF-7 inhibition rate was 70.23±4.54%.

These data demonstrate that at fixed exposure times and concentrations, lingonberry extract had the greatest inhibitory effect on HepG2 cells.

#### Inhibitory effect of various vaccinium berries on HepG2 proliferation

**Fig 2:**
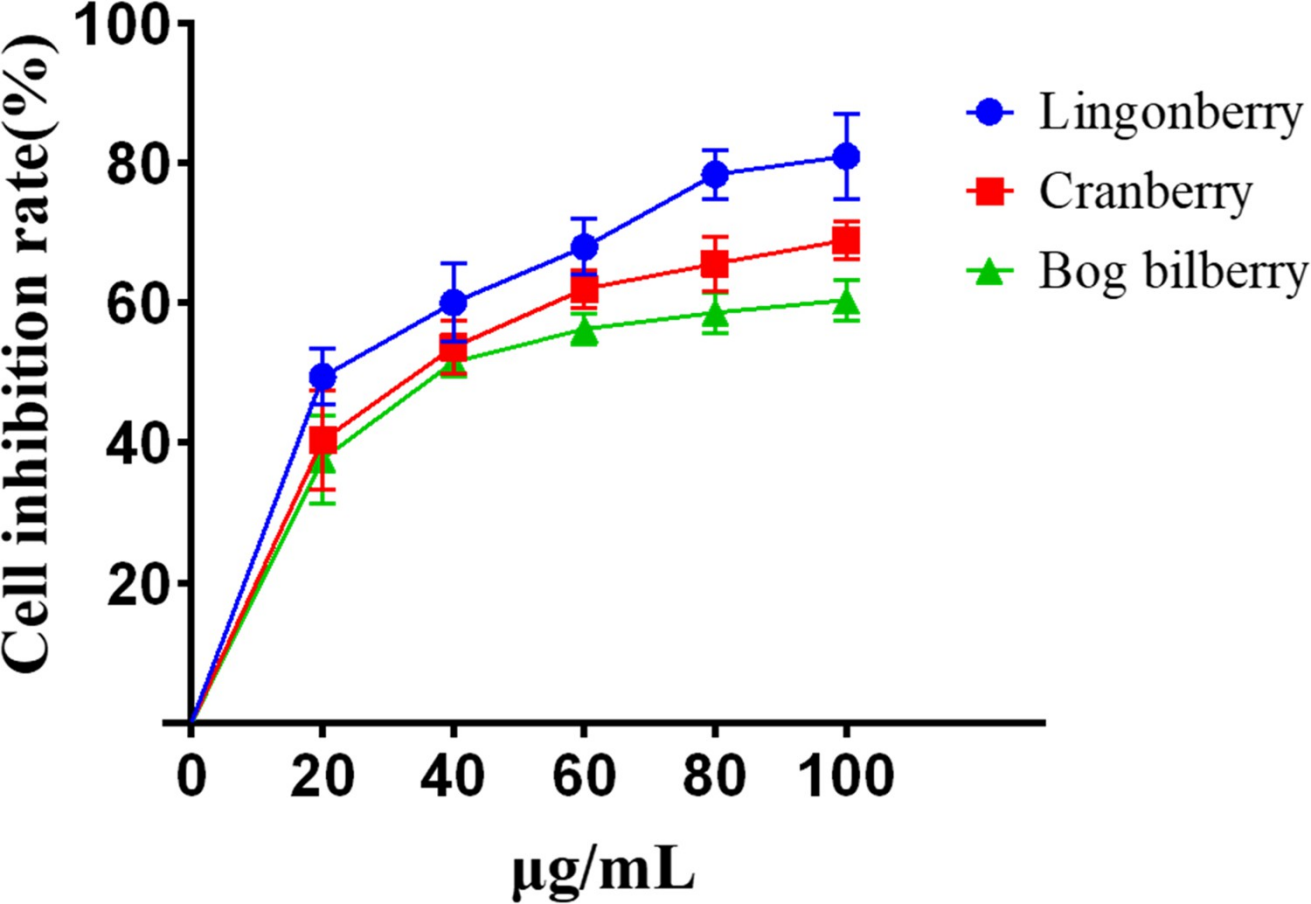
Inhibition rates of various *Vaccinium* berry extracts on proliferation of HepG2 cells.

CCK-8 assay (Fig 2) shows that different *Vaccinium* berry extracts had significant inhibitory effects on the proliferation of HepG2 cells and this effect increased at higher concentrations.

When cells were treated with extracts for 48 h, lingonberry had an inhibition rate of 78.34±3.55% and IC_50_ of 22.62 μg/mL at 80 μg/mL. Inhibition rate increased at higher concentrations, but not significantly above that at 80 μg/mL. At 100 μg/mL, the inhibition rate was 80.89±6.05%.

When the concentration of cranberry extract reached 60 μg/mL, inhibition rate and IC_50_ were 61.98±2.74% and 33.06 μg/mL, respectively. With increasing concentration the inhibition rate increased, but not significantly above that at 60 μg/mL, reaching 68.93±2.67% at 100 μg/mL.

Bog bilberry extract achieved an inhibition rate of 51.47±2.01% and an IC_50_ of 42.55 μg/mL at 40 μg/mL. Inhibition increased as concentration increased, but not significantly above that at 40 μg/mL (inhibition rate reached 60.35±2.94% at 100 μg/mL). Of the three *Vaccinium* berry extracts, lingonberry exhibited the strongest inhibitory effect on HepG2 cells.

#### Inhibitory effect of lingonberry treatment duration on HepG2 proliferation

**Fig 3:**
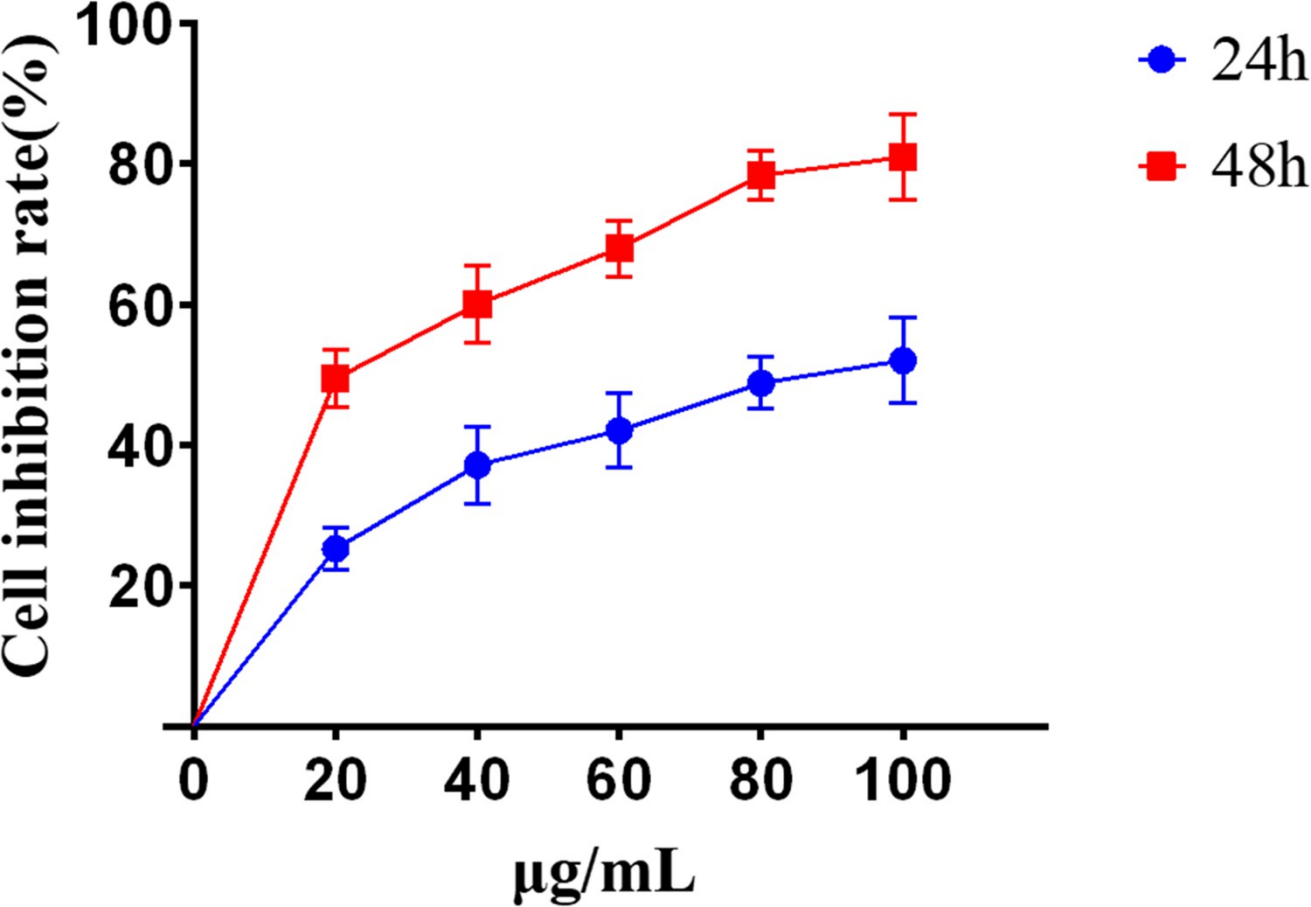
Inhibition rates of lingonberry extracts on proliferation of HepG2 cells treated for 24 h and 48 h.

The CCK-8 assay data in Fig 3 show that the inhibitory effect of lingonberry extracts on the proliferation of HepG2 cells increased with extended exposure time. After 24 h treatment at 100 μg/mL, the inhibition rate and IC_50_ reached 51.99±6.16% and 88.39 μg/mL, respectively. After 48 h at 100 μg/mL, inhibition and IC_50_ reached 80.89±6.05% and 22.62 μg/mL, respectively. These data demonstrate that lingonberry extract inhibits HepG2 cells in a dose- and time-dependent manner.

#### Effect of lingonberry extracts on HepG2 cell apoptosis

**Fig 4:**
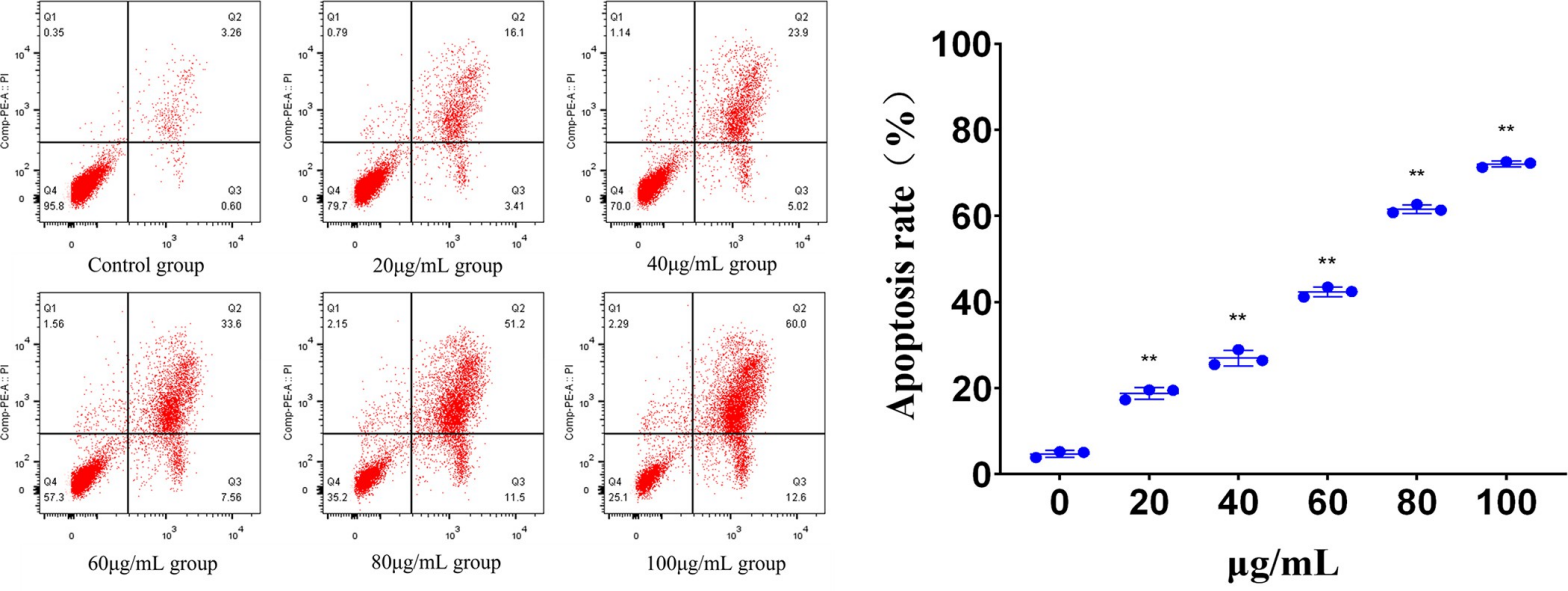
Effect of lingonberry extract on the apoptosis rate of HepG2 cells. (**a**) Effect of extract concentration on apoptosis. (**b**) Analysis of cell apoptosis rates in lingonberry treatment and control groups (** *p* < 0.01 compared to the control group).

The effect of lingonberry extracts on HepG2 cell apoptosis was evaluated using flow cytometry. In Fig 4, quadrant 2 (Q2) represents late apoptosis and Q3 early apoptosis. Exposure to *Vaccinium vitis-idaea* L. increased the number of early and late apoptotic HepG2 cells, with a greater increase in late apoptotic cells as the extract concentration increased (Fig 4A). The apoptosis rate in all lingonberry-treated groups was considerably higher than the control group (Fig 4B). Treatment with 100 μg/mL extract for 48 h produced an apoptosis rate of 71.22±3.58%, suggesting that the inhibitory effect of lingonberry extract on HepG2 cells is associated with the induction of cell apoptosis.

#### Effect of lingonberry extract on HepG2 cell cycle

**Fig 5:**
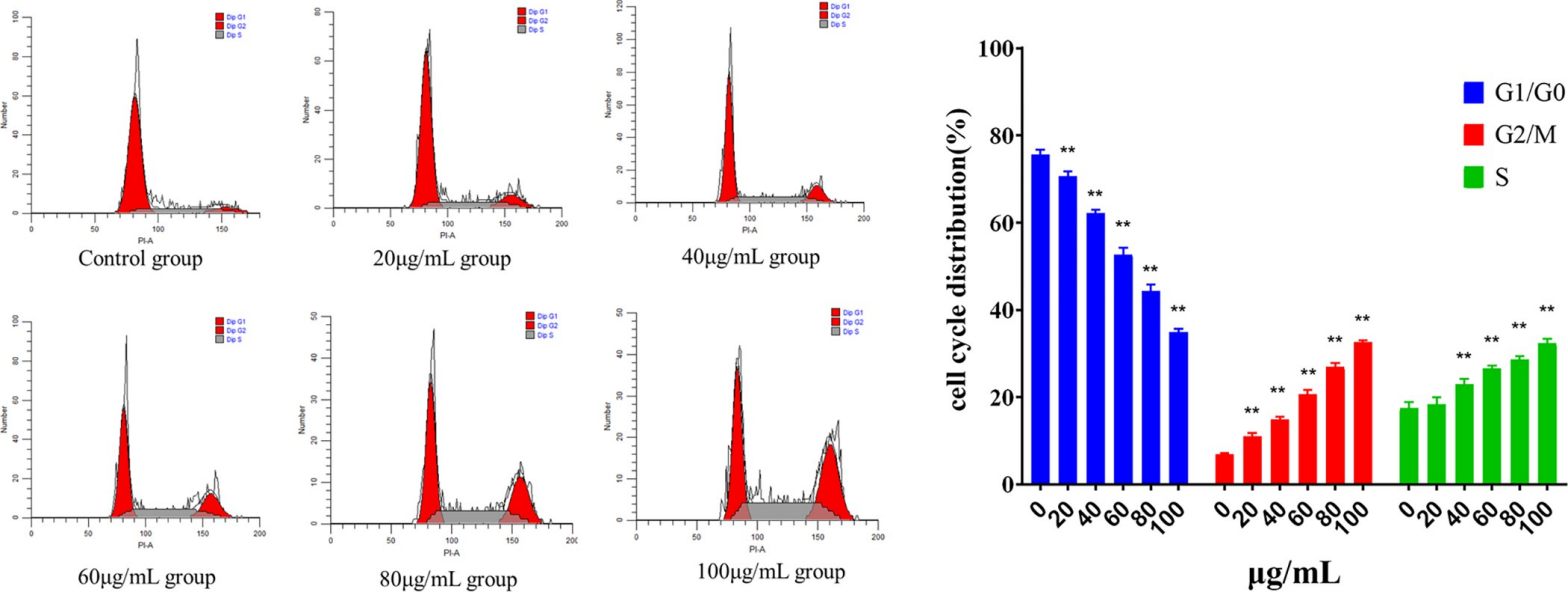
Effect of lingonberry extract on the apoptosis cycle of HepG2 cells. (**a**) Effect of lingonberry extract concentration on the cell cycle. (**b**) Analysis of cell apoptosis cycle in lingonberry treatment and control groups (* *p* < 0.05, ** *p* < 0.01 compared to the control group).

Fig 5 illustrates the effect of lingonberry extract on the HepG2 cell cycle. As the concentration of extract increased, apoptosis of HepG2 cells increased, with the number of cells at G1/G0 phase declining and cells at S and G2/M phases gradually increasing. At 40, 60, 80, and 100 μg/mL, the numbers of cells at S and G2/M phases were significantly higher than in the control group. This suggests that lingonberry extract arrests the cell cycle predominantly at the G2/M and S phases.

#### Effect of lingonberry extracts on HepG2 cell migration and invasion

**Fig 6:**
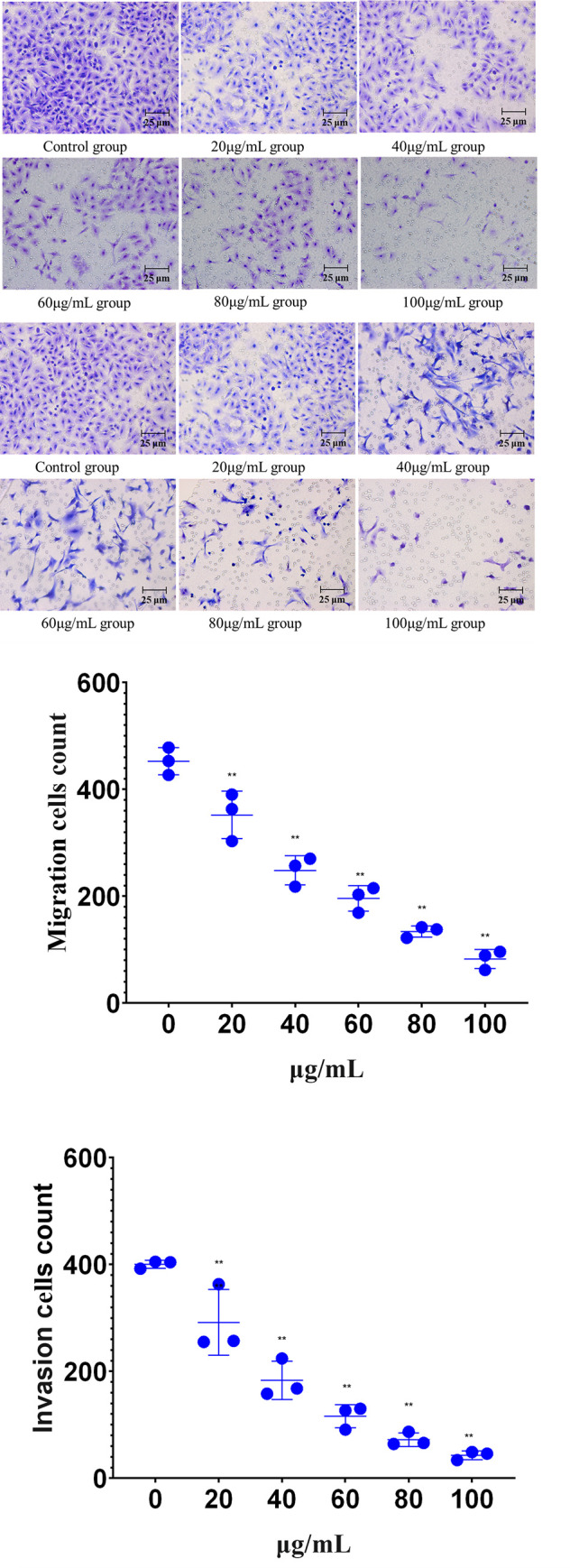
Effects of lingonberry extract on the migration and invasion of HepG2 cells. (**a**) Effects of extract concentration on the migration of HepG2 cells. (**b**) Effects of extract concentration on the invasion of HepG2. (**c**) Analysis of cell migration in lingonberry treatment and control groups. (**d**) Analysis of cell invasion in lingonberry treatment and control groups (** *p* < 0.01 compared to the control group).

A transwell assay was conducted to observe the effects of lingonberry extract on the migration and invasion capabilities of HepG2 cells. Compared with the control group (Fig 6), the number of migrating and invading extract-treated HepG2 cells decreased significantly as extract concentration increased, indicating that lingonberry extract significantly inhibits the migration and invasion of HepG2 cells, proportional to the extract concentration.

#### Effect of lingonberry extract on expression of CXCL3 in HepG2 cells

**Fig 7:**
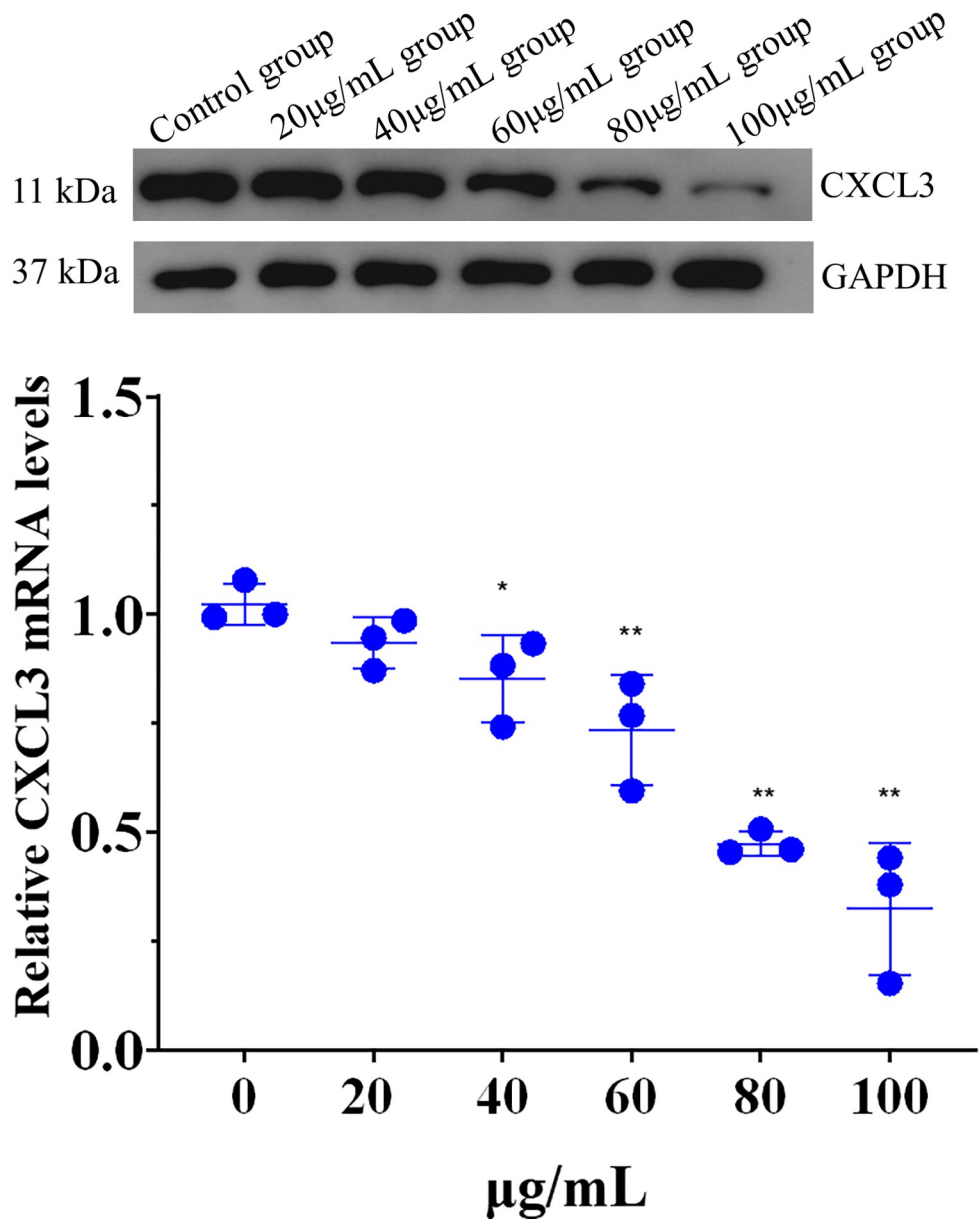
Effects of lingonberry extract on the protein expression of CXCL3. (**a**) Protein expression of CXCL3 detected by western blotting. (**b**) Relative expression of CXCL3 determined by real-time PCR (* *p* < 0.05, ** *p* < 0.01 compared to the control group).

Western blotting showed that the expression of CXCL3 decreased as lingonberry extract concentration increased compared with the control group (Fig 7A). Thus, lingonberry extract affects the protein expression of CXCL3 in a dose-dependent manner.

Fig 7B shows that CXCL3 expression declined in all lingonberry extract treatment groups compared with the control (0 μg/mL). Expression of CXCL3 was significantly suppressed in the 40 μg/mL group and strongly significantly suppressed in the 60, 80, and 100 μg/mL groups. There was no significant difference between the 80 and 100 μg/mL groups, so 80 μg/mL was selected for subsequent experiments.

**Fig 8:**
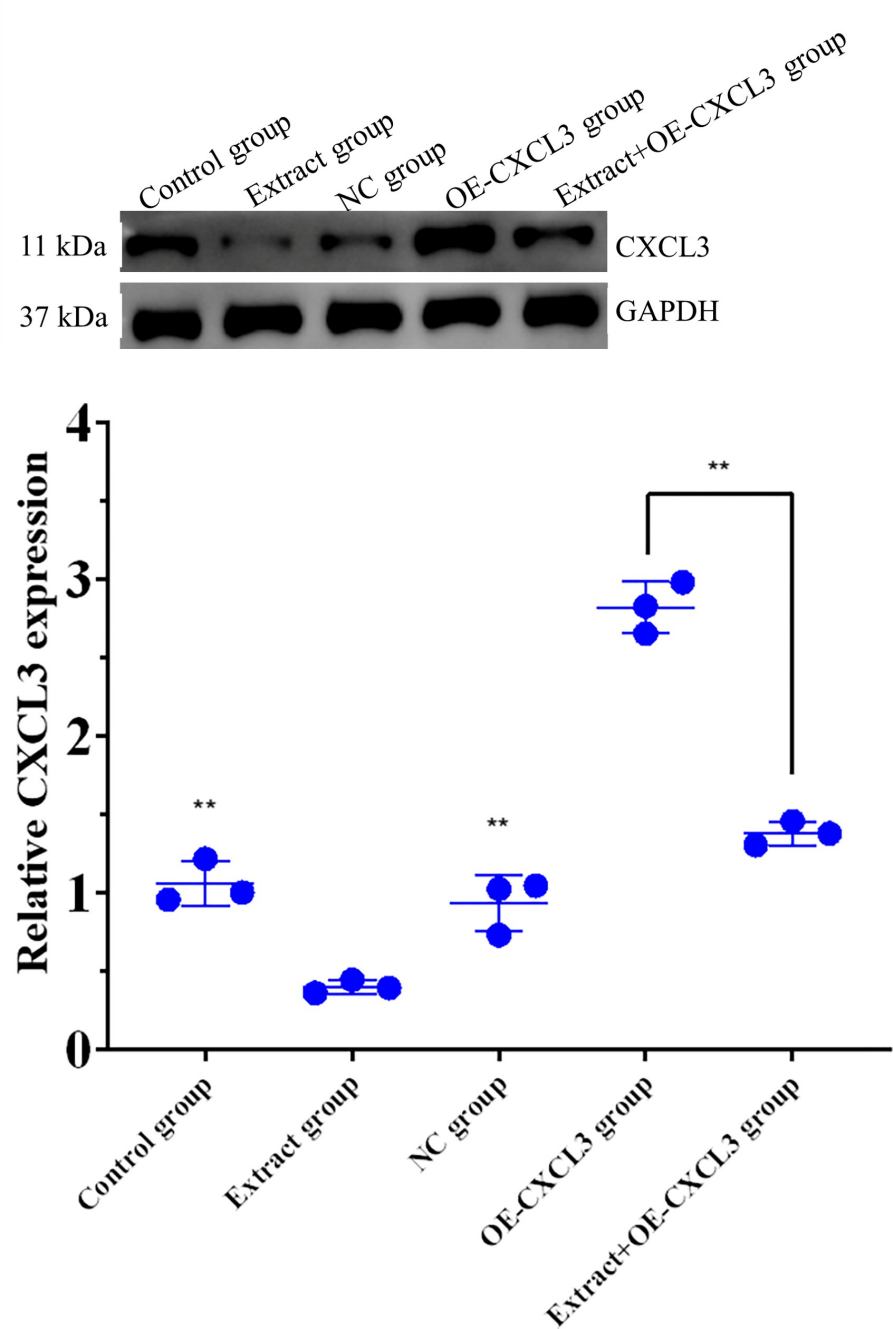
Expression of CXCL3 in control, extract, and transfection groups. (**a**) Protein expression of CXCL3 detected by western blotting. (**b**) Relative expression of CXCL3 determined by real-time PCR. (** *p* < 0.01 compared to the extract group).

Western blotting and PCR show that CXCL3 expression in the 80 μg/mL extract group was significantly lower than in the control and NC groups. CXCL3 expression was significantly lower in the extract+OE-CXCL3 group than the OE-CXCL3 group (Fig 8A and 8B), demonstrating that lingonberry extract inhibits the expression of CXCL3 protein.

#### Effect of lingonberry extract on proliferation, apoptosis, migration, and invasion of HepG2 cells via regulation of CXCL3 expression

**Fig 9:**
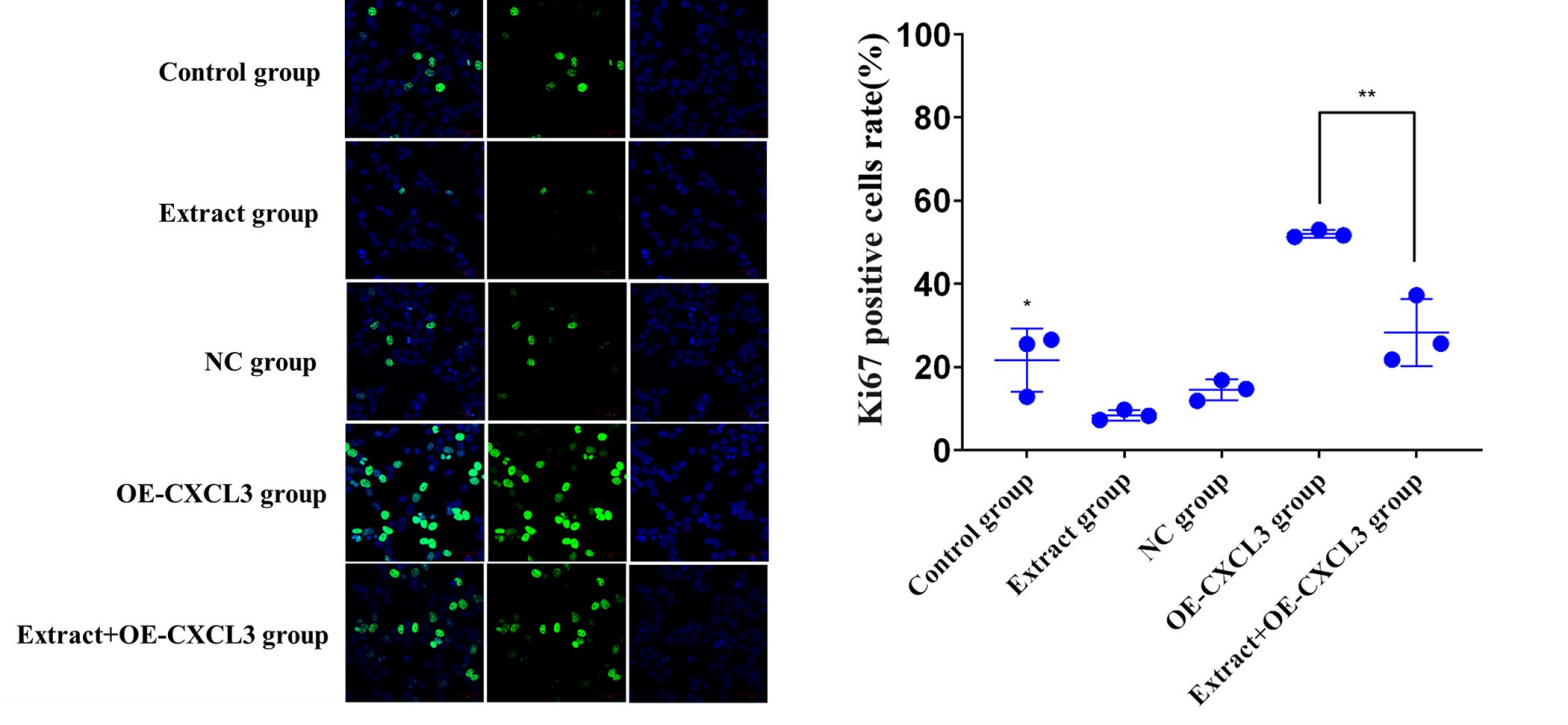
Proliferation of HepG2 cells in control, extract, and transfection groups. (**a**) ×400 magnification fluorescence micrographs of the effects of treatment on HepG2 cell proliferation. (**b**) Ki-67 positive cell rates (* *p* < 0.05, ** *p* < 0.01 compared to the extract group).

Ki-67 assay data (Fig 9) show that the Ki-67 positive cell rate in the extract group was significantly lower than the control group, and the extract+OE-CXCL3 group was significantly lower than the OE-CXCL3 group. This indicates that lingonberry extract can inhibit cell proliferation by regulating the expression of CXCL3.

**Fig 10:**
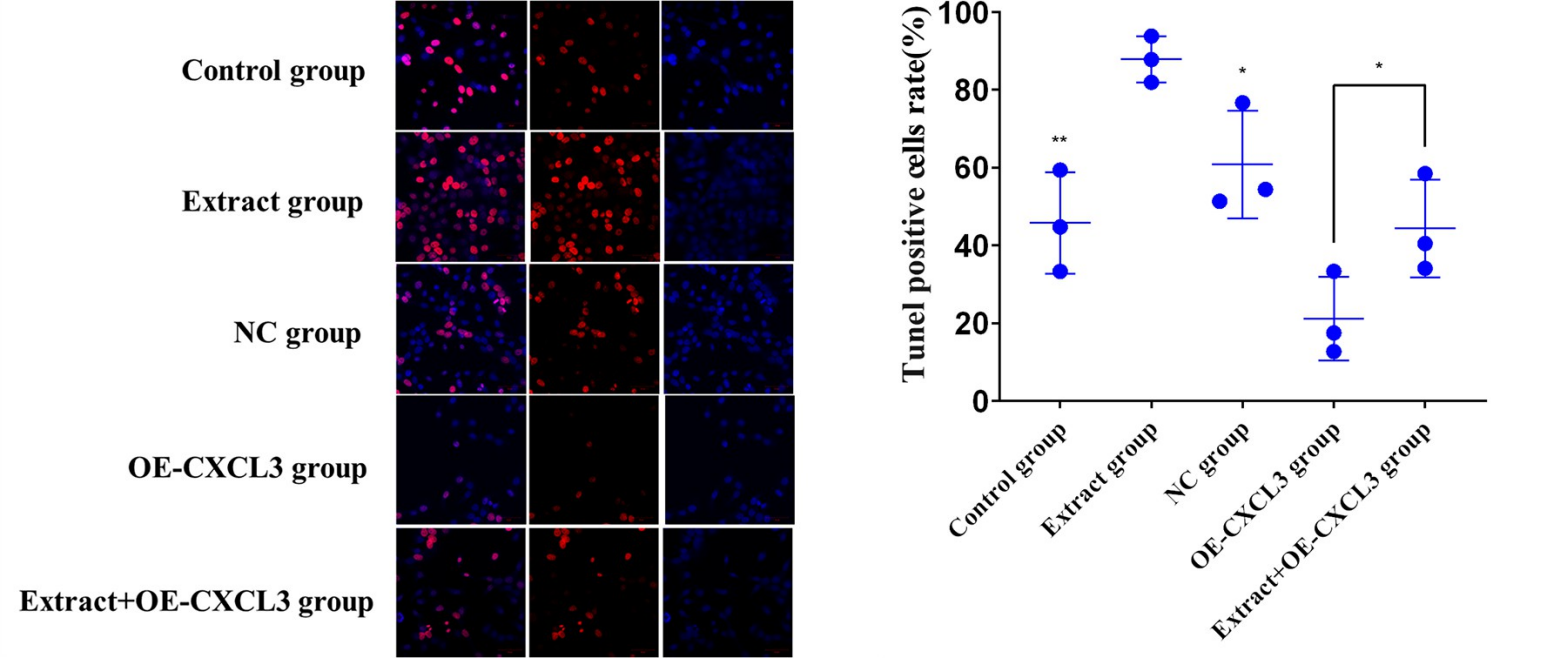
Apoptosis of HepG2 cells in control, extract, and transfection groups. (**a**) ×400 magnification light micrographs of the effects of treatment on HepG2 cell apoptosis. (**b**) TUNEL apoptosis positive cell rates (* *p* < 0.05, ** *p* < 0.01 compared to the extract group).

TUNEL assay data (Fig 10) show that the TUNEL positive cell rate was significantly higher in the extract group than the control and NC groups, and the extract+OE-CXCL3 group was significantly higher than the OE-CXCL3 group. This indicates that lingonberry extract can accelerate the apoptosis of HepG2 cells by regulating the expression of CXCL3.

**Fig 11:**
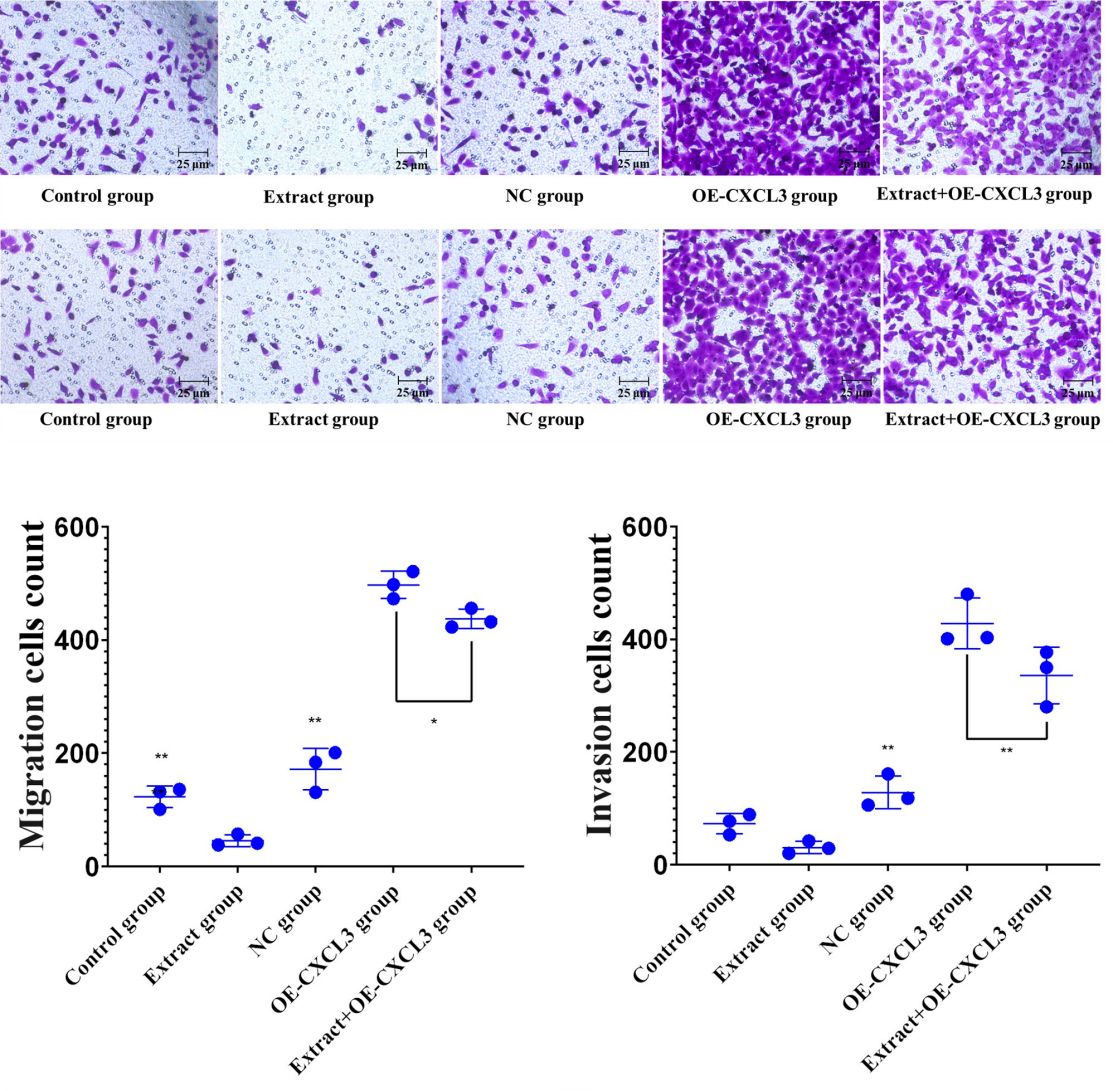
Migration and invasion of HepG2 cells in control, extract, and transfection groups. (**a**) ×200 magnification micrographs of the effects of treatment on HepG2 cell migration. (**b**) ×200 magnification micrographs of the effects of treatment on HepG2 cell invasion. (**c**) Migration cell counts. (**d**) Invasion cell counts. (* *p* < 0.05, ** *p* < 0.01 compared to the extract group).

Transwell assay data (Fig 11A and 11C) show that the migration cell count was significantly lower in the extract group than the control and NC groups, and the extract+OE-CXCL3 group was significantly lower than the OE-CXCL3 group. This indicates that lingonberry extract can inhibit cell migration by regulating the expression of CXCL3. Transwell data (Fig 11B and 11D) also show that the invasion cell count in the extract group was significantly lower than the NC group, and the extract+OE-CXCl3 group was significantly lower than the OE-CXCL3 group. These results demonstrate that lingonberry extract inhibits both migration and invasion of HepG2 cells by regulating CXCL3 expression.

#### Lingonberry extract composition

**Fig 12:**
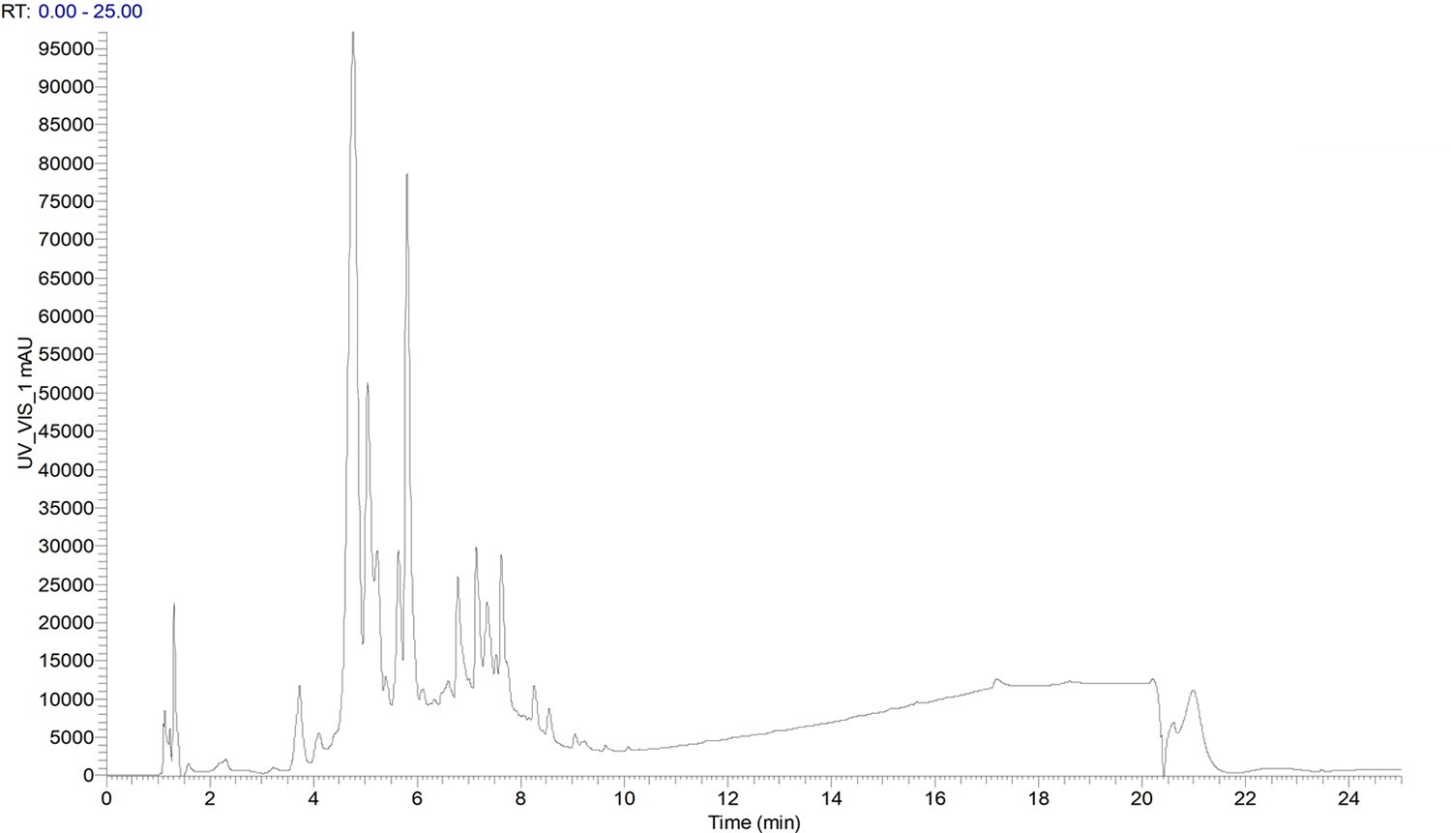
UV-Vis spectrum at 280 nm of lingonberry extract.

These components were present in the following proportions (Fig 12): 195.88 μg/mg cyanidin-3-O-glucoside chloride (37.58%), 57.13 μg/mg kaempferol 3-O-arabinoside (10.96%), 23.56 μg/mg epicatechin (4.52%), 22.67 μg/mg chlorogenic acid (4.35%), 19.96 μg/mg catechinic acid (3.83%), 8.03 μg/mg isoquercitrin (1.54%), 5.47 μg/mg 4-hydroxycinnamic acid (1.05%), 5.37 μg/mg cyanidin chloride (1.03%), 4.43 μg/mg 2,3-dihydroxybenzoic acid (0.85%), 2.87 μg/mg quercetin (0.55%), 1.88 μg/mg D-(-)-quinic acid (0.36%), 1.20 μg/mg caffeic acid (0.96%), 0.83 μg/mg ferulic acid (0.16%), 0.63 μg/mg oleanolic acid (0.12%), and 0.17 μg/mg ursolic acid (0.03%).

## Discussion

Lingonberry extract contains a large number of polyphenols, comprised mainly of cyanidin-3-O-glucoside chloride, kaempferol 3-O-arabinoside, catechinic acid, quercetin, and chlorogenic acid. Catechinic acid, quercetin, and phenolic acids such as caffeic acid and ferulic acid can suppress the growth of cancer cells [29]. This study demonstrates that lingonberry extract rich in these functional components had different degrees of inhibitory effects on liver, colon and breast cancer cells, with the strongest effect on the proliferation of HepG2 cells. The *Vaccinium* berries bog bilberry, cranberry and lingonberry all inhibited the growth of HepG2 hepatoma cells, with lingonberry having the strongest effect. Lingonberry contains the highest proportion of procyanidins (818 μg/mg) compared with cranberry (690 μg/mg) and bog bilberry (255 μg/mg), whilst (+)-catechin is also highest in lingonberry (584 μg/mg), followed by cranberry (417 μg/mg) and bog bilberry (70 μg/mg) [30]. These differences are likely to account for the different inhibitory effects on cancer cell proliferation. Phenolic acids and flavonoids in berries can penetrate the cell membrane of cancer cells, producing a strong antiproliferative effect [31]. As the concentration of lingonberry increased, these various components synergistically enhanced their inhibitory effect on HepG2 cells. However, it remains to be experimentally verified which specific components are responsible for suppressing HepG2 growth.

Under normal physiological and pathological conditions, apoptosis is a spontaneous process of cell death that can be activated by various stimuli. One mechanism of action of many antitumor drugs is to induce abnormal cell apoptosis, thereby interfering with tumor growth [32–35]. This study demonstrates that lingonberry extract can suppress the proliferation of HepG2 cells in a dose-dependent manner by inducing apoptosis *in vitro*. It produces a higher response in late apoptosis than early apoptosis, with the late apoptosis rate rising significantly as lingonberry extract concentration increases. This suggests that lingonberry extract has a stronger inductive effect on the late apoptosis of HepG2 cells and has a positive dose-response correlation.

The effect of lingonberry extract on the HepG2 cell cycle was also studied. The proportion of treated HepG2 cells at G2/M and S phases gradually increased, indicating cell cycle arrest and proving that lingonberry extract suppresses HepG2 by regulating the cell cycle. *Aristotelia chilensis* (maqui) berry arrests the cycle of Ishikawa cells at G2/M phase [36]. Cyanidin 3-sambubioside weakens cell viability and arrests the cell cycle of human leukemia-60 cells at G2/M phase [37]. Kaempferol induces G2/M cell cycle arrest and promotes apoptosis of A2780/CP70 human ovarian cancer cells [38]. Quercetin inhibits the proliferation of HT29 colon cancer cells and, in combination with Adriamycin, can arrest the G2/M phase [39]. These reports are in line with the present study indicating that the active ingredients of lingonberry extract largely determine how it suppresses cell proliferation. Lingonberry extract was also proven to suppress HepG2 cell migration and invasion, preventing cells from passing through the chamber. Higher lingonberry doses had stronger inhibitory effects on HepG2 migration and invasion.

CXCL3 is closely related to tumor growth and can affect the proliferation, invasion, and migration of tumor cells by activating relevant cell pathways in combination with their receptors [40]. CXCL3 and CXCL8 are the key genes that affect the diagnosis and prognosis of colon adenocarcinoma [41]. Overexpressed CXCL3 can heighten the risk of tumorigenesis in prostate cancer and cervical cancer [12, 14]. An increasing number of studies suggest that CXCL3 can serve as a novel predictor of tumor progression. In this study, western blotting and PCR analysis demonstrated that lingonberry extract significantly reduces the expression of CXCL3 in HepG2 cells. To further elucidate the relationship between CXCL3 expression and cell proliferation, apoptosis, migration, and invasion, experiments were conducted after cell transfection using Ki-67, TUNEL, and transwell assays. Data indicated that inhibition of CXCL3 expression promoted apoptosis of HepG2 cells and inhibited proliferation, migration, and invasion of HepG2 cells. Thus, lingonberry extract can inhibit the proliferation, migration, and invasion of HepG2 cells and promote their apoptosis by regulating the expression of CXCL3. This study provides a new direction for the treatment of hepatoma (utilizing the inhibitory effects of lingonberry extract on the chemokine CXCL3), offers new insight into the study of lingonberry’s antihepatoma properties, and provides a basis for the development of pilot antitumor drugs.

## Conclusions

Lingonberry extract contains a variety of functional components. It can inhibit the growth, proliferation, apoptosis, migration, and invasion of human hepatoma HepG2 cells by inhibiting the expression of chemokine CXCL3. This finding provides a foundation for the potential development of new anti-liver cancer drugs.

## Supporting information

S1 Fig Effects of lingonberry extract on the protein expression of CXCL3. (a) Protein expression of CXCL3 detected by western blotting.
(PDF)

S2 Fig Expression of CXCL3 in control, extract, and transfection groups. (a) Protein expression of CXCL3 detected by western blotting.
(PDF)

S1 Dataset (XLSX)

## References

[1] Sayiner M, Golabi P, Younossi ZM. Disease Burden of Hepatocellular Carcinoma: A Global Perspective. Dig Dis Sci. 2019; 64:910–7. doi: 10.1007/s10620-019-05537-2 . 30835028

[2] Omata M, Cheng A-L, Kokudo N, Kudo M, Lee JM, Jia J, et al. Asia-Pacific clinical practice guidelines on the management of hepatocellular carcinoma: a 2017 update. Hepatol Int. 2017; 11:317–70. Epub 2017/06/15. doi: 10.1007/s12072-017-9799-9 . 28620797 PMC5491694

[3] Kowalska K. Lingonberry (Vaccinium vitis-idaea L.) Fruit as a Source of Bioactive Compounds with Health-Promoting Effects-A Review. Int J Mol Sci. 2021; 22. Epub 2021/05/12. doi: 10.3390/ijms22105126 . 34066191 PMC8150318

[4] Ștefănescu B-E, Călinoiu LF, Ranga F, Fetea F, Mocan A, Vodnar DC, et al. Chemical Composition and Biological Activities of the Nord-West Romanian Wild Bilberry (Vaccinium myrtillus L.) and Lingonberry (Vaccinium vitis-idaea L.) Leaves. Antioxidants (Basel). 2020; 9. Epub 2020/06/05. doi: 10.3390/antiox9060495 . 32517130 PMC7346130

[5] Prakash MD, Stojanovska L, Feehan J, Nurgali K, Donald EL, Plebanski M, et al. Anti-cancer effects of polyphenol-rich sugarcane extract. PLoS One. 2021; 16:e0247492. Epub 2021/03/10. doi: 10.1371/journal.pone.0247492 . 33690618 PMC7946306

[6] Cásedas G, Les F, Gómez-Serranillos MP, Smith C, López V. Anthocyanin profile, antioxidant activity and enzyme inhibiting properties of blueberry and cranberry juices: a comparative study. Food Funct. 2017; 8:4187–93. doi: 10.1039/c7fo01205e . 29038797

[7] Liu J, Zhang W, Jing H, Popovich DG. Bog bilberry (Vaccinium uliginosum L.) extract reduces cultured Hep-G2, Caco-2, and 3T3-L1 cell viability, affects cell cycle progression, and has variable effects on membrane permeability. J Food Sci. 2010; 75:H103–7. doi: 10.1111/j.1750-3841.2010.01546.x . 20492295

[8] Armania N, Yazan LS, Ismail IS, Foo JB, Tor YS, Ishak N, et al. Dillenia Suffruticosa extract inhibits proliferation of human breast cancer cell lines (MCF-7 and MDA-MB-231) via induction of G2/M arrest and apoptosis. Molecules. 2013; 18:13320–39. Epub 2013/10/29. doi: 10.3390/molecules181113320 . 24172241 PMC6269718

[9] Shu C-W, Weng J-R, Chang H-W, Liu P-F, Chen J-J, Peng C-C, et al. Tribulus terrestris fruit extract inhibits autophagic flux to diminish cell proliferation and metastatic characteristics of oral cancer cells. Environ Toxicol. 2021; 36:1173–80. Epub 2021/03/10. doi: 10.1002/tox.23116 . 33751830

[10] Gordon JR, Li F, Zhang X, Wang W, Zhao X, Nayyar A. The combined CXCR1/CXCR2 antagonist CXCL8(3–74)K11R/G31P blocks neutrophil infiltration, pyrexia, and pulmonary vascular pathology in endotoxemic animals. J Leukoc Biol. 2005; 78:1265–72. Epub 2005/10/04. doi: 10.1189/jlb.0805458 . 16204619

[11] Singh S, Wu S, Varney M, Singh AP, Singh RK. CXCR1 and CXCR2 silencing modulates CXCL8-dependent endothelial cell proliferation, migration and capillary-like structure formation. Microvasc Res. 2011; 82:318–25. Epub 2011/07/01. doi: 10.1016/j.mvr.2011.06.011 . 21749879 PMC3215896

[12] Gui S-L, Teng L-C, Wang S-Q, Liu S, Lin Y-L, Zhao X-L, et al. Overexpression of CXCL3 can enhance the oncogenic potential of prostate cancer. Int Urol Nephrol. 2016; 48:701–9. Epub 2016/02/02. doi: 10.1007/s11255-016-1222-2 . 26837773

[13] Pei X, Chen S-W, Long X, Zhu S-Q, Qiu B-Q, Lin K, et al. circMET promotes NSCLC cell proliferation, metastasis, and immune evasion by regulating the miR-145-5p/CXCL3 axis. Aging (Albany NY). 2020; 12:13038–58. Epub 2020/07/02. doi: 10.18632/aging.103392 . 32614785 PMC7377868

[14] Qi Y-L, Li Y, Man X-X, Sui H-Y, Zhao X-L, Zhang P-X, et al. CXCL3 overexpression promotes the tumorigenic potential of uterine cervical cancer cells via the MAPK/ERK pathway. J Cell Physiol. 2020; 235:4756–65. Epub 2019/10/30. doi: 10.1002/jcp.29353 . 31667838

[15] Takenaga K, Akimoto M, Koshikawa N, Nagase H. Cancer cell-derived interleukin-33 decoy receptor sST2 enhances orthotopic tumor growth in a murine pancreatic cancer model. PLoS One. 2020; 15:e0232230. Epub 2020/04/27. doi: 10.1371/journal.pone.0232230 . 32340025 PMC7185704

[16] Vilkickyte G, Raudone L, Petrikaite V. Phenolic Fractions from Vaccinium vitis-idaea L. and Their Antioxidant and Anticancer Activities Assessment. Antioxidants (Basel). 2020; 9. Epub 2020/12/11. doi: 10.3390/antiox9121261 . 33322638 PMC7763140

[17] Bujor O-C, Ginies C, Popa VI, Dufour C. Phenolic compounds and antioxidant activity of lingonberry (Vaccinium vitis-idaea L.) leaf, stem and fruit at different harvest periods. Food Chem. 2018; 252:356–65. Epub 2018/01/09. doi: 10.1016/j.foodchem.2018.01.052 . 29478554

[18] Kylli P, Nohynek L, Puupponen-Pimiä R, Westerlund-Wikström B, Leppänen T, Welling J, et al. Lingonberry (Vaccinium vitis-idaea) and European cranberry (Vaccinium microcarpon) proanthocyanidins: isolation, identification, and bioactivities. J Agric Food Chem. 2011; 59:3373–84. Epub 2011/03/03. doi: 10.1021/jf104621e . 21370878

[19] Dróżdż P, Šėžienė V, Wójcik J, Pyrzyńska K. Evaluation of Bioactive Compounds, Minerals and Antioxidant Activity of Lingonberry (Vaccinium vitis-idaea L.) Fruits. Molecules. 2017; 23. Epub 2017/12/26. doi: 10.3390/molecules23010053 . 29278401 PMC5943965

[20] Eid HM, Ouchfoun M, Brault A, Vallerand D, Musallam L, Arnason JT, et al. Lingonberry (Vaccinium vitis-idaea L.) Exhibits Antidiabetic Activities in a Mouse Model of Diet-Induced Obesity. Evid Based Complement Alternat Med. 2014; 2014:645812. Epub 2014/06/10. doi: 10.1155/2014/645812 . 25013446 PMC4072050

[21] Isaak CK, Petkau JC, Blewett H, O K, Siow YL. Lingonberry anthocyanins protect cardiac cells from oxidative-stress-induced apoptosis. Can J Physiol Pharmacol. 2017; 95:904–10. Epub 2017/04/06. doi: 10.1139/cjpp-2016-0667 . 28384410

[22] Madduma Hewage S, Prashar S, Debnath SC, O K, Siow YL. Inhibition of Inflammatory Cytokine Expression Prevents High-Fat Diet-Induced Kidney Injury: Role of Lingonberry Supplementation. Front Med (Lausanne). 2020; 7:80. Epub 2020/03/27. doi: 10.3389/fmed.2020.00080 . 32292787 PMC7119336

[23] Onali T, Kivimäki A, Mauramo M, Salo T, Korpela R. Anticancer Effects of Lingonberry and Bilberry on Digestive Tract Cancers. Antioxidants (Basel). 2021; 10. Epub 2021/05/26. doi: 10.3390/antiox10060850 . 34073356 PMC8228488

[24] Hoornstra D, Vesterlin J, Pärnänen P, Al-Samadi A, Zlotogorski-Hurvitz A, Vered M, et al. Fermented Lingonberry Juice Inhibits Oral Tongue Squamous Cell Carcinoma Invasion In Vitro Similarly to Curcumin. In Vivo. 2018; 32:1089–95. doi: 10.21873/invivo.11350 . 30150430 PMC6199607

[25] Li X, Zou K, Gou J, Du Q, Li D, He X, et al. Effect of baicalin-copper on the induction of apoptosis in human hepatoblastoma cancer HepG2 cells. Med Oncol. 2015; 32:72. Epub 2015/02/19. doi: 10.1007/s12032-015-0527-9 . 25694047

[26] Weng J, Ren Q, Li Z, Wang W, Guan J. CXCL3 overexpression affects the malignant behavior of oral squamous cell carcinoma cells via the MAPK signaling pathway. J Oral Pathol Med. 2021; 50:902–10. Epub 2021/08/25. doi: 10.1111/jop.13234 . 34392586

[27] Genç H, Hazur J, Karakaya E, Dietel B, Bider F, Groll J, et al. Differential Responses to Bioink-Induced Oxidative Stress in Endothelial Cells and Fibroblasts. Int J Mol Sci. 2021; 22. Epub 2021/02/26. doi: 10.3390/ijms22052358 . 33652991 PMC7956320

[28] Liu L, Yang L, Chang H, Chen Y-N, Zhang F, Feng S, et al. CP‑31398 attenuates endometrial cancer cell invasion, metastasis and resistance to apoptosis by downregulating MDM2 expression. Int J Oncol. 2019; 54:942–54. Epub 2019/01/09. doi: 10.3892/ijo.2019.4681 . 30628640 PMC6365028

[29] Stagos D, Amoutzias GD, Matakos A, Spyrou A, Tsatsakis AM, Kouretas D. Chemoprevention of liver cancer by plant polyphenols. Food Chem Toxicol. 2012; 50:2155–70. Epub 2012/04/11. doi: 10.1016/j.fct.2012.04.002 . 22521445

[30] Määttä-Riihinen KR, Kähkönen MP, Törrönen AR, Heinonen IM. Catechins and procyanidins in berries of vaccinium species and their antioxidant activity. J Agric Food Chem. 2005; 53:8485–91. doi: 10.1021/jf050408l . 16248542

[31] Rupasinghe V, Neir SV, Parmar I. Polyphenol characterization, anti-oxidant, anti-proliferation and anti-tyrosinase activity of cranberry pomace. FFHD. 2016; 6:754. doi: 10.31989/ffhd.v6i11.292

[32] Badroon NA, Abdul Majid N, Alshawsh MA. Antiproliferative and Apoptotic Effects of Cardamonin against Hepatocellular Carcinoma HepG2 Cells. Nutrients. 2020; 12. Epub 2020/06/12. doi: 10.3390/nu12061757 . 32545423 PMC7353428

[33] Rajasekaran D, Manoharan S, Silvan S, Vasudevan K, Baskaran N, Palanimuthu D. Proapoptotic, anti-cell proliferative, anti-inflammatory and anti-angiogenic potential of carnosic acid during 7,12 dimethylbenzaanthracene-induced hamster buccal pouch carcinogenesis. Afr J Tradit Complement Altern Med. 2012; 10:102–12. Epub 2012/10/01. doi: 10.4314/ajtcam.v10i1.14 . 24082331 PMC3746363

[34] Zhang Q-Y, Yue X-Q, Jiang Y-P, Han T, Xin H-L. FAM46C is critical for the anti-proliferation and pro-apoptotic effects of norcantharidin in hepatocellular carcinoma cells. Sci Rep. 2017; 7:396. Epub 2017/03/24. doi: 10.1038/s41598-017-00313-6 . 28341836 PMC5428258

[35] Al Refaey HR, Newairy A-SA, Wahby MM, Albanese C, Elkewedi M, Choudhry MU, et al. Manuka honey enhanced sensitivity of HepG2, hepatocellular carcinoma cells, for Doxorubicin and induced apoptosis through inhibition of Wnt/β-catenin and ERK1/2. Biol Res. 2021; 54:16. Epub 2021/05/28. doi: 10.1186/s40659-021-00339-1 . 34049576 PMC8161992

[36] Mena J, Elgueta E, Espinola-Gonzales F, Cardenas H, Orihuela PA. Hydroethanolic Extracts of the Aristotelia Chilensis (Maqui) Berry Reduces Cellular Viability and Invasiveness in the Endometrial Cancer Cell Line Ishikawa. Integr Cancer Ther. 2021; 20:15347354211007560. doi: 10.1177/15347354211007560 . 33926283 PMC8113921

[37] Tsai T-C, Huang H-P, Chang K-T, Wang C-J, Chang Y-C. Anthocyanins from roselle extract arrest cell cycle G2/M phase transition via ATM/Chk pathway in p53-deficient leukemia HL-60 cells. Environ Toxicol. 2017; 32:1290–304. Epub 2016/07/22. doi: 10.1002/tox.22324 . 27444805

[38] Gao Y, Yin J, Rankin GO, Chen YC. Kaempferol Induces G2/M Cell Cycle Arrest via Checkpoint Kinase 2 and Promotes Apoptosis via Death Receptors in Human Ovarian Carcinoma A2780/CP70 Cells. Molecules. 2018; 23. Epub 2018/05/05. doi: 10.3390/molecules23051095 . 29734760 PMC6065264

[39] Atashpour S, Fouladdel S, Movahhed TK, Barzegar E, Ghahremani MH, Ostad SN, et al. Quercetin induces cell cycle arrest and apoptosis in CD133(+) cancer stem cells of human colorectal HT29 cancer cell line and enhances anticancer effects of doxorubicin. Iran J Basic Med Sci. 2015; 18:635–43. 26351552 PMC4556754

[40] Wang H, Wang T, Dai L, Cao W, Ye L, Gao L, et al. Effects of CXCL3 on migration, invasion, proliferation and tube formation of trophoblast cells. Placenta. 2018; 66:47–56. Epub 2018/05/26. doi: 10.1016/j.placenta.2018.05.004 . 29884302

[41] Zhao Q-Q, Jiang C, Gao Q, Zhang Y-Y, Wang G, Chen X-P, et al. Gene expression and methylation profiles identified CXCL3 and CXCL8 as key genes for diagnosis and prognosis of colon adenocarcinoma. J Cell Physiol. 2020; 235:4902–12. Epub 2019/11/10. doi: 10.1002/jcp.29368 . 31709538

