# Supplementary figures and images for: Inhibitory effect of lingonberry extract on HepG2 cell proliferation, apoptosis, migration, and invasion
Source: PLoS One. 2022 Jul 8;17(7):e0270677. doi: 10.1371/journal.pone.0270677 (PMC9269931; doi:10.1371/journal.pone.0270677)

S7 (a) Figure

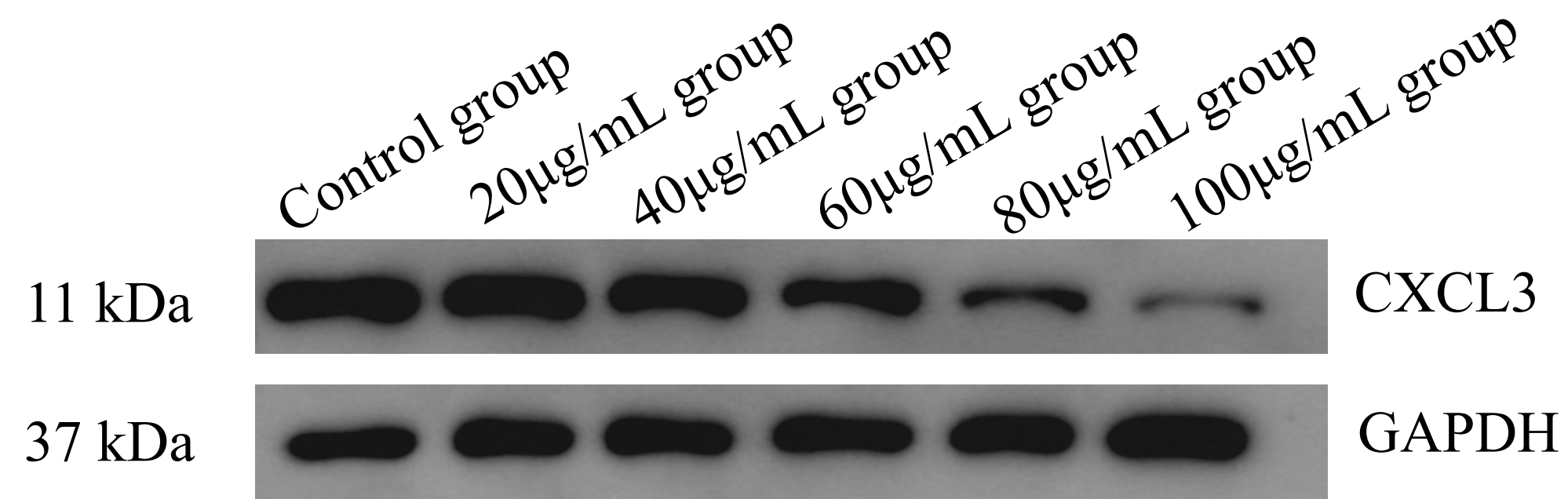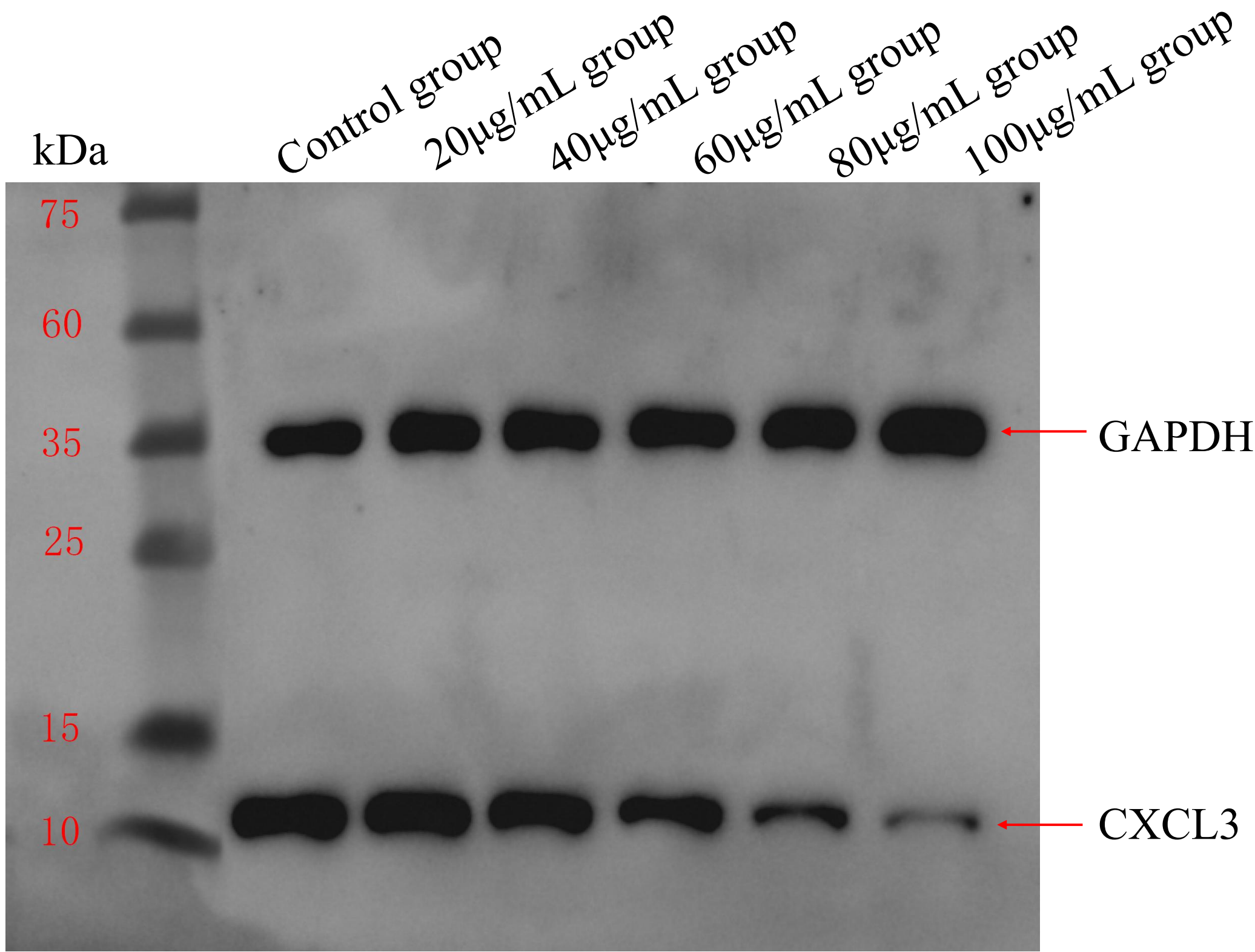

Supplement: S1 Fig — (a) Protein expression of CXCL3 detected by western blotting. (PDF) [file pone.0270677.s001.pdf]

S8(a) Figure

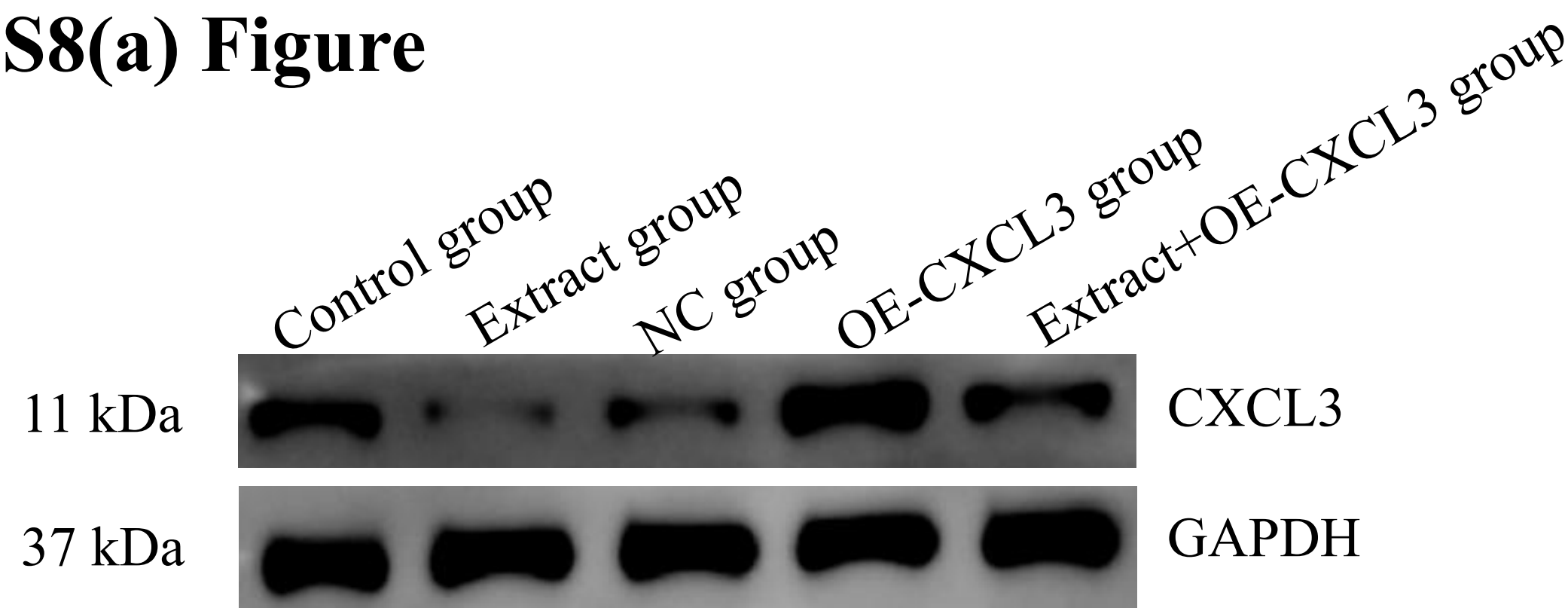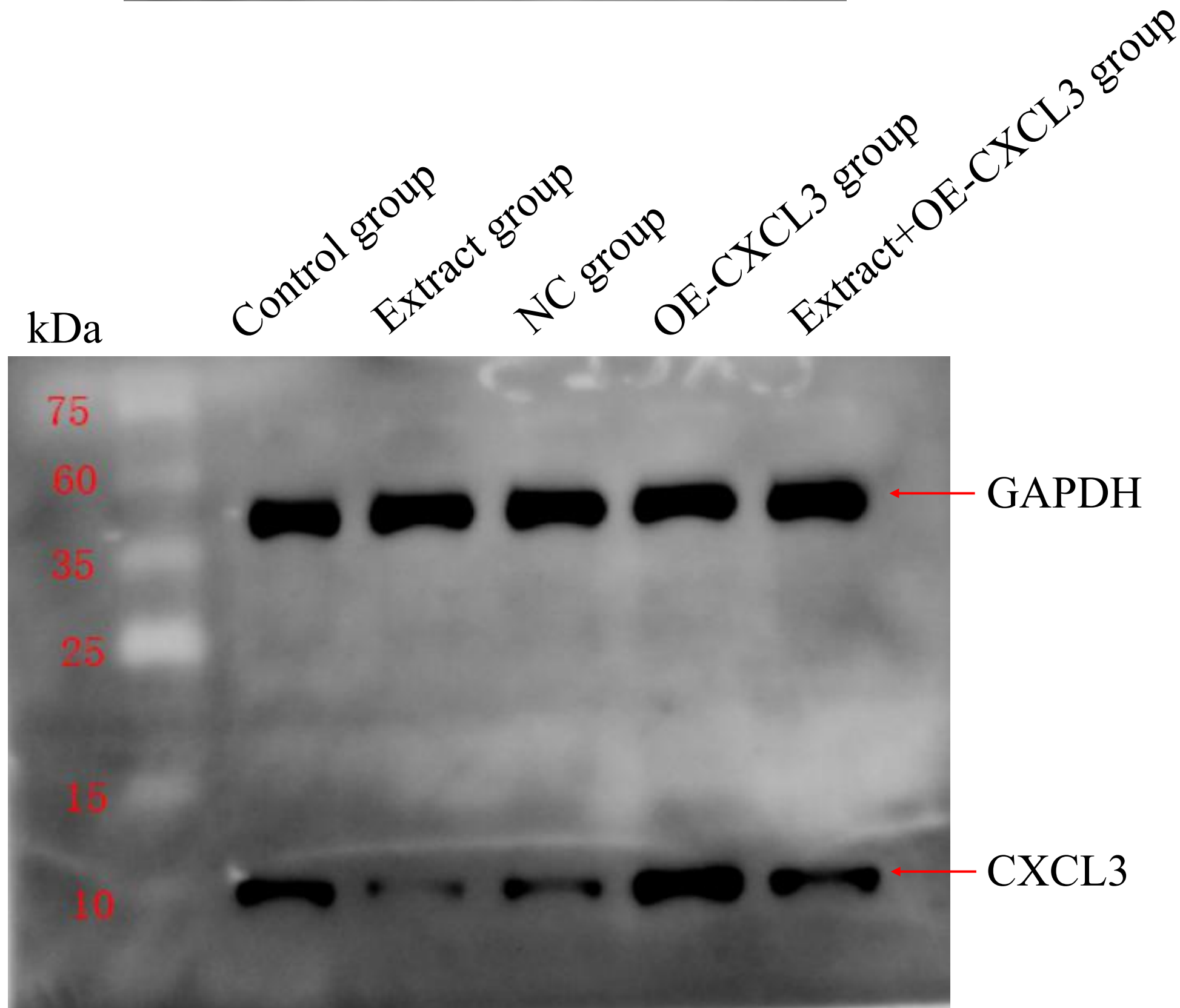

Supplement: S2 Fig — (a) Protein expression of CXCL3 detected by western blotting. (PDF) [file pone.0270677.s002.pdf]
